# Trends in acute respiratory infection and associated factors among under-5 children in Ethiopia

**DOI:** 10.1186/s12887-025-06100-x

**Published:** 2026-01-19

**Authors:** Ermias Tadesse Beyene, Seungman Cha, Ducksu Seo, Yan Jin

**Affiliations:** 1 Department of Human Ecology and Technology, Graduate School of Advanced Convergence, Handong Global University, Pohang, 37554 Republic of Korea; 2 Department of Global Development and Entrepreneurship, Graduate School of Global Development and Entrepreneurship, Handong Global University, Pohang, 37554 South Korea; 3 School of Spatial Environmental System Engineering, Handong Global University, 558 Handongro, Bukgu, Pohang, 37554 Republic of Korea; 4 Department of Microbiology, Dongguk University College of Medicine, Gyeongju, Korea

**Keywords:** Acute respiratory infection, Under-5 children, Trend, Ethiopia, Child health

## Abstract

**Background:**

Acute respiratory infections (ARI) are the leading cause of death in children under five. In 2021, an estimated five million children died before turning five. Sub-Saharan Africa and South Asian countries bear burdens of 56% and 26% of the global mortality, respectively. However, studies regarding trends in the prevalence of ARI and its associated factors over a wide period are scarce. Hence, we aimed to assess the trends and factors associated with the prevalence of ARI in Ethiopia over the past 16 years.

**Methods:**

We used the 2000, 2005, 2011, and 2016 EDHS datasets. We conducted a trend analysis of the past 16 years and applied a multilevel logistics regression analysis to identify the individual-, household- and cluster-level factors affecting ARI among under-5 children.

**Results:**

At the national level, the prevalence of ARI in under-5 children in Ethiopia fell from 11.50% in 2000 to 9.37% in 2016. However, the temporal trend in the prevalence of ARI per region showed inconsistent patterns. From 2011 to 2016, the Benishangul-Gumuz, Gambela, Somali, Afar, and Amhara regions and Dire Dawa city administration showed a reduction in ARI prevalence, whereas the remaining regions and Addis Ababa city administration showed an increase in ARI prevalence. Our model, with individual-, household- and cluster-level factors, was found to be the best fitting model for exploring ARI prevalence-affecting factors. Compared with children aged < 12 months, children aged 24–35 months were less likely to develop ARI (adjusted odds ratio [AOR] = 0.68, 95% confidence interval [CI]: 0.59–0.79, *p* < 0.001). Children who were wasted were more likely to develop ARI than those who were not wasted, with an AOR of 1.18 (95% CI: 1.00–1.39, *p* = 0.04).

**Conclusion:**

This study identified inconsistent trends in the prevalence of ARI at the regional level of Ethiopia from 2000 to 2016. To the best of our knowledge, this is the first nationwide trend analysis of ARI in Ethiopia. Further research is warranted to identify the reasons for the substantial differences in the temporal trend of ARI by region in Ethiopia.

**Supplementary Information:**

The online version contains supplementary material available at 10.1186/s12887-025-06100-x.

## Introduction

An estimated five million children lost their lives before their fifth birthday in 2021 [1]. Sub-Saharan Africa and South Asia shoulder the heaviest burden, accounting for 56% and 26% of total mortality, respectively [1]. According to the 2019 World Health Organization (WHO) report, acute respiratory infection (ARI), diarrhea, malaria, and preterm birth complications are among the leading causes of death in children under the age of 5 years, many of which are preventable and treatable [2]. ARI is one of the most common childhood illnesses, contributing greatly to the morbidity and mortality of under-5 children [3]. Sub-Saharan Africa and central and southern Asian countries account for 52% of the global under-5 population, and these two regions contributed to more than 80% of the 5.2 million-under-5 mortality in 2019 [2]. The United Nations Children’s Fund (UNICEF) report also states that these regions are highly affected, accounting for approximately 70% of ARI [4]. Five countries, including Ethiopia, contributed to half of all the under-5 mortality in 2019 [2]. If the current trend continues, 54 nations will soon fall short of meeting the sustainable development goal (SDG) target for under-5 child mortality [2].

ARI is an infection that can occur at various sites along the respiratory system, disrupting an individual’s normal breathing patterns [5]. Acute lower respiratory infections affect the airways from the trachea and bronchi to the bronchioles and alveoli, while acute upper respiratory infections impact the airways from the nose to the vocal cords in the larynx [6, 7]. According to research, “Acute respiratory infection is identified by coughing accompanied by rapid, shallow breathing and can often lead to death due to complications with other childhood illnesses.” [8, 9]. Pneumonia is considered one of the most severe consequences of ARI in children under the age of five [10, 11]. According to the Global Burden of Diseases (GBD) study in 2015, ARI was the deadliest disease in Ethiopia, accounting for 10% of deaths each year, followed by diarrhea which accounts for about 8% of deaths in the country yearly [12]. Recent studies indicate that Ethiopia has attained remarkable achievements in improving child survival, with under-5 mortality dropping by two-thirds [13].

Many cross-sectional studies have identified individual factors that contribute to the prevalence of ARI [14–20]. Individual-level factors include child characteristics such as age [14–18] and sex [19], and maternal characteristics such as maternal age [14, 16], work status [14, 16], and educational status [19]. Studies on the cluster-level factors for ARI were scarce [20, 21]. In addition, there is a paucity of studies on trends in the prevalence of ARI over time in Ethiopia. Hence, this study aimed to assess the trend in the prevalence of ARI and its associated factors at individual-, household- and cluster-level over sixteen years in Ethiopia.

## Materials and methods

### Study area

This study was conducted in Ethiopia, located in northeastern Africa, also known as the horn of Africa. The country is divided into eleven regions: Afar; Amhara; Benishangul-Gumuz; Gambella; Harai; Oromiya; Southern Nations, Nationalities, and People’s Region (SNNPR); Somali; Tigray; and the recently added Sidama and South-West regions, and two city administrations: Addis Ababa and Dire Dawa. The Sidama Zone became a region in November 2019, and the South-West region was established on November 23, 2021. Ethiopia shares boundaries with Eritrea in the north, Sudan and South Sudan in the west, Kenya and Somalia in the south, and Djibouti and Somalia in the east.

### Data source: Ethiopian demographic and health surveys 2000–2016

We use the EDHS for four consecutive survey years (2000, 2005, 2011, and 2016). The EDHS is a population-based cross-sectional study conducted across the country. The four DHS in Ethiopia surveyed 15,367, 14,070, 16,515, and 15,683 women of reproductive age (15–49 years) in 2000, 2005, 2011, and 2016, respectively. Sample selection for secondary survey analysis was conducted using a two-stage sampling procedure. In the first stage, Enumeration Areas (EAs) for the surveys were selected from nine regions and two city administrations. An EA is a geographic area covering an average of 150–200 households and the type of residence can be urban or rural.

The EAs were chosen from a list authorized by the Ethiopian Central Statistics Agency as a sampling framework for the Population and Household Census (PHC). The second stage involved selecting a specified number of households from each cluster using a systematic sampling technique. A cluster refers to either an EA or a segment of an EA in case of large EAs.

**Fig. 1:**
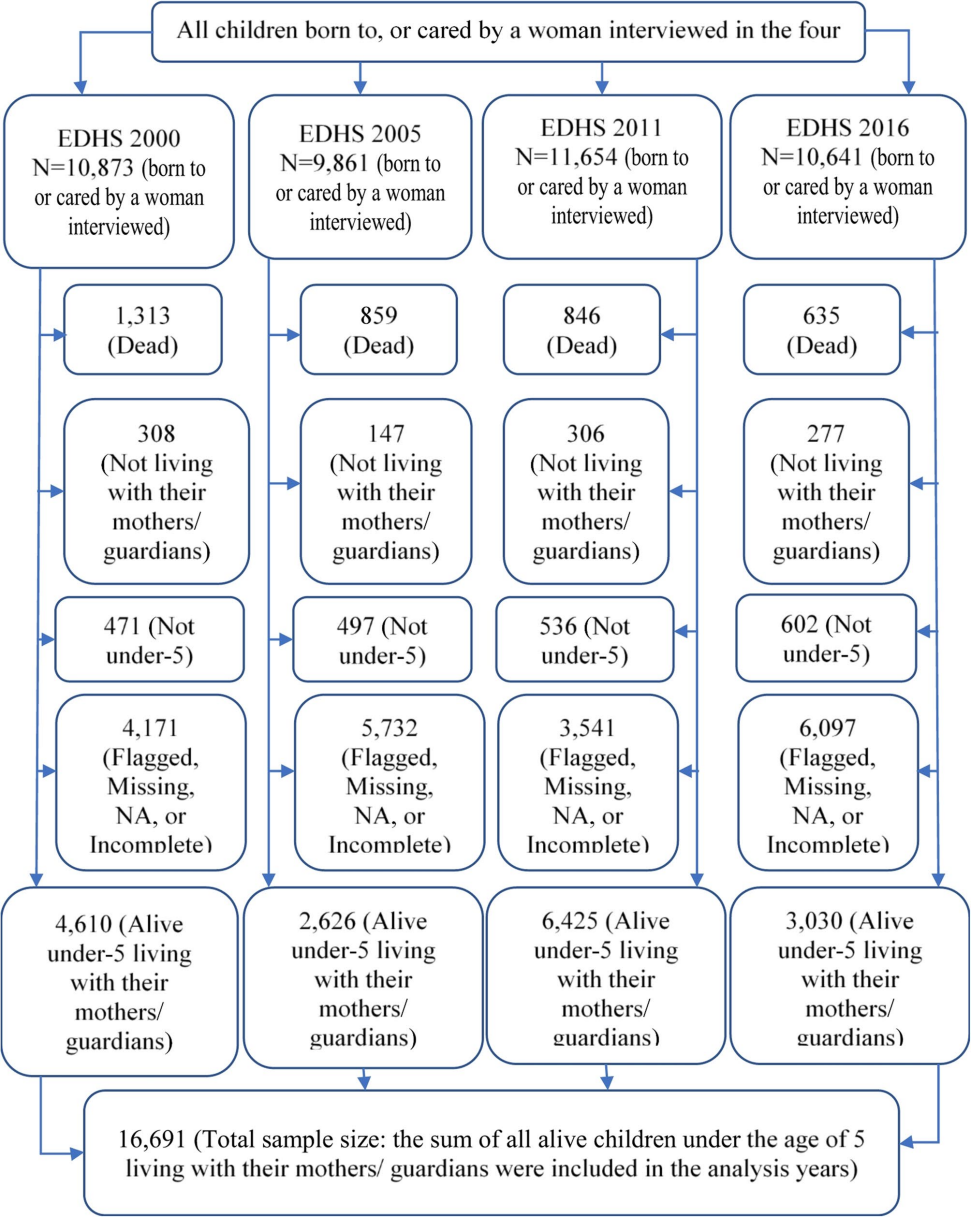
Flow diagram of sample selection for this study

Details of the sampling method/design can be found in the EDHS reports for successive survey years [22–25]. All women aged 15–49 years who were eligible for the surveys were selected for the women’s questionnaire, which collected data on ARI and other issues. All women aged 15–49 years who had at least one child in the five years before the survey were eligible for participation. We extracted our working dataset from the merged dataset of children and household records for each survey year. We filtered for the under-5 and still alive children. The sample size for this study consisted of 36232 under-5 children (8781, 8358, 9966, and 9127 from the 2000, 2005, 2011, and 2016 surveys, respectively (Fig. 1). As the EDHS data contained many missing values across some variables, we excluded flagged, missing, and NA cases. The data was cleaned using SPSS statistical software (version 24; IBM Corporation, Armonk, NY); the statistical analysis and graph plotting was performed using R Statistical Software (version 4.1.2; R Core Team 2021).

### Statistical analysis

The primary outcome of interest (indicator) was ARI. This indicator is a binary variable being “Yes” if the child/children of the participating mother suffered from a cough during the last two weeks before the survey, accompanied by shortness of breath or rapid breathing problems, and “No” if the child/children of the participating mother did not suffer from a cough during that period. The DHS provide a weighting factor. Weighting was used because of possible disparities in response rates and the non-proportional allocation of the sample to different regions, including urban and rural areas. Therefore, all figures in the Results section were obtained using weighted samples to account for variances related to the study design, stratification, and sampling processes. A complex survey sampling technique was used to analyze the weighted data. We have estimated a 95% confidence interval for ARI prevalence as well as percentage point of arithmetic difference of ARI prevalence between the two different time periods incorporating weighing factors and using R Statistical Software (version 4.1.2; R Core Team 2021).

#### Dependent variable

The outcome of interest for measurement in our study was the dichotomous variable of the prevalence of ARI among under-5 children.

$$\mathrm{Yi}=\{1,\;\mathrm{if}\;\mathrm{the}\;\mathrm i^{\mathrm{th}}\;\mathrm{child}\;\mathrm{suffered}\;\mathrm{from}\;\mathrm{ARI}:\;0,\;\mathrm{otherwise}\}$$

#### Independent variables

We classified the independent variables into two categories: 

1. The individual (child-related and household-related variables)
2. Cluster-related variables

The child-related variables were the child’s age, vaccination status, and wasting. Household-related variables were maternal education, marital status, religion of the mother, cooking fuel type, total number of living children, and maternal body mass index (BMI). Both the child and household related variables are directly extracted from the EDHS, with some undergoing simple transformations.

#### Cluster-related variables

Place of residence, survey years, cluster-level media access, and cluster-level access to electricity were cluster-level variables. The place of residence indicated whether the respondent lived in an urban or rural area, about which we referred to a study suggesting that it determines a person’s access to services, information about health, and other aspects of life [24]. The two variables place of residence and survey year, were directly taken from the dataset. However, they were transformed, aggregated, recategorized, and used to record individual-level variables in order to obtain cluster-level media access, and cluster-level access to electricity. To determine cluster-level media access, the variables frequency of reading newspapers or magazines, frequency of listening to the radio, and frequency of watching television were each transformed into new binary variables (1 for a frequency of at least once a week, 0 otherwise). These variables were then combined into a single binary variable called “media access”. Lastly the variable media access was aggregated at the cluster level by calculating the mean and transforming into a new binary variable (1 for a frequency greater than the mean, 0 otherwise). A similar approach was used to generate cluster-level access to electricity.

#### Trend in the ARI prevalence

To explore the temporal trend of ARI prevalence during the study period, we broadly divided the study period into three phases: 2000–2005, 2005–2011, and 2011–2016. Descriptive statistics were used to show trends for each specific study variable. However, we focused on the regional-level trends. Both the weighted and unweighted results are shown.

#### Factors associated with Acute Respiratory Infection prevalence

To identify the major individual-, household-, and cluster-level factors that determine the prevalence of ARI in under-5 children, we used multi-level mixed-effect logistic regression for the 2000–2016 data. We built four successive models and assessed their Akaike Information Criterion (AIC) and Bayesian Information Criterion (BIC) values to select the best-fit model. The goodness of fit of the adjusted final model was estimated using the AIC and BIC statistics and checked using a log-likelihood test in comparison to the preceding models. The smaller the values of AIC and BIC, the better the model fit. The first model was the intercept only (null model), and no explanatory variables were included to show the total variance between clusters. The second model was a fixed effects model that encompasses all individual- and household-level variables, in which we put variables of significant association with ARI with a p-value < 0.25. The third model was a random-effects model with only cluster-level variables. The final (fourth) model was the mixed-effect model, which holds both individual- and household-level variables (fixed effect) and cluster-level variables (random effect). Statistical significance was set at *p* < 0.05.

The model we used is as follows:-.

$$\begin{aligned}\log&\lbrack{\mathrm\pi}_{\mathrm{ijk}}/(1-{\mathrm\pi}_{\mathrm{ijk}})\rbrack=\mathrm{Logit}({\mathrm\pi}_{\mathrm{ijk}})={\mathrm\beta}_0\\&+\mathrm\beta^{\mathrm T}{\mathrm X}_{\mathrm{ijk}}+\mathrm\gamma ^{\mathrm T}{\mathrm Z}_{\mathrm{jk}}+\mathrm\delta^{\mathrm T}{\mathrm W}_{\mathrm k}+{\mathrm u}_{\mathrm k}+{\mathrm u}_{\mathrm{jk}}\end{aligned}$$

where π_ijk_ = P(Y_ijk_ = 1) is the probability of the ARI occurring for the i-th child in the j-th cluster in the k-th survey year. $\:{\beta\:}_{0}$ is the is the overall fixed intercept (the overall average of a child acquiring ARI). $\:\beta\:$ is a vector of fixed effects coefficients for the child-level predictors *X*_*ijk*_. γ is a vector of fixed effects coefficients for the household-level predictors *Z*_*jk*_. $\delta$ is a vector of fixed effects coefficients for the cluster-level predictors *W*_*k*_. *u*_*k*_ is the random effect at the cluster level, assumed to follow a normal distribution with mean 0 and variance $\:{}_{u}^{2}$, i.e., u_k_∼N(0, $\:{}_{u}^{2}$). *u*_*jk*_ is the random effect at the household level within each cluster, assumed to follow a normal distribution with mean 0 and variance $\:{}_{u}^{2}$, i.e., *u*_*jk*_ ∼N(0, $\:{}_{u}^{2}$).

To assess individual- and household-level variation, we analyzed the fixed effect using the adjusted odds ratio (AOR) along with 95% confidence intervals (CI). For cluster-level variation (random effect), we computed the intraclass correlation coefficient (ICC) value to examine the clustering effect and the degree to which the unexplained variance of the null model is explained by the cluster-level factors using the following statistical formula:

$$\:ICC=\frac{{}^{2}a}{{}^{2}a+{}^{2}b}$$

where $\:{}^{2}a$ indicates the cluster-level, and $\:{}^{2}b$ does individual- and household-level variance, respectively. Individual- and household-level variance $\:{}^{2}b$ is a fixed value of $\:\frac{{}^{2}}{3}$.

## Results

### Background characteristics of children and their mothers

The total number of under-5 children who were included in the analysis was 16,691. The combined dataset revealed that a greater proportion of under-5 children were 24–35 months old, accounting for 25.28%. Approximately Half (51.06%) of the under-5 children were male. Approximately 90.01% of the under-5 children were not wasted. A greater proportion (55.85%) of the children’s mothers (respondents) had no education, and the majority (93.69%) of respondents were in a union. Across the survey years, most respondents were Orthodox Christians (except in 2016), and the proportion of Orthodox believers declined in successive years. Table 1 presents the characteristics of children, their respondents (child mothers/guardians) households over the four EDHS years.

**Table 1:** Background characteristics of under-5 children and households of the Ethiopian demographic and health survey years 2000, 2005, 2011, and 2016 (*N* = 16691)

Variables	Survey Year				
	2000 (*N* = 4610)	2005 (*N* = 2626)	2011 (*N* = 6425)	2016 (*N* = 3030)	Pooled (2000–2016) (*N* = 16691)
	*n*(%)	*n*(%)	*n*(%)	*n*(%)	*n*(%)
Child’s age					
< 12 months	623 (13.15)	304 (12.03)	1998 (31.53)	735 (26.00)	3660 (20.68)
12–23 months	811 (18.37)	470 (17.04)		1025 (33.53)	2306 (17.24)
24–35 months	923 (19.45)	543 (21.35)	1343 (19.86)	1270 (40.47)	4079 (25.28)
36–47 months	1147 (25.53)	658 (25.21)	1577 (24.80)		3382 (18.89)
48–59 months	1106 (23.51)	651 (24.37)	1507 (23.81)		3264 (17.92)
Child’s sex					
Male	2343 (50.60)	1345 (50.62)	3331 (52.14)	1534 (50.88)	8553 (51.06)
Female	2267 (49.40)	1281 (49.38)	3094 (47.86)	1496 (49.12)	8138 (48.94)
Wasting					
Yes	525 (10.13)	292 (10.56)	645 (8.28)	422 (11.01)	1884 (10.00)
No	4085 (89.87)	2334 (89.44)	5780 (91.72)	2608 (88.99)	14,807 (90.01)
Mother’s educational level					
No education	3871 (83.48)	2115 (81.31)	4771 (72.8)	2103 (68.41)	12,860 (55.85)
Primary	519 (12.40)	390 (15.69)	1479 (24.87)	747 (27.91)	3135 (40.76)
Secondary & higher	220 (4.11)	121 (3.01)	175 (2.33)	180 (3.68)	696 (3.40)
Marital Status					
Never in Union	21 (0.38)	11 (0.22)	23 (0.43)	20 (0.86)	75 (0.47)
In a Union	4291 (92.98)	2465 (94.17)	5996 (93.02)	2877 (94.57)	15,629 (93.69)
Not in a Union	298 (6.64)	150 (5.61)	406 (6.56)	133 (4.57)	987 (5.85)
Type of cooking fuel					
Electricity/LPG/Natural gas/Biogas	7 (0.02)	12 (0.11)	16 (0.12)	42 (0.98)	77 (0.31)
Kerosene	154 (1.05)	52 (0.54)	34 (0.14)	2 (0.22)	242 (0.43)
Coal/lignite/charcoal	79 (0.62)	42 (0.62)	224 (2.89)	137 (2.30)	482 (1.61)
Firewood/straw	3902 (82.93)	2397 (92.19)	5958 (91.12)	2754 (91.10)	15,011 (89.34)
Dung	468 (15.38)	123 (6.53)	193 (5.73)	95 (5.62)	879 (8.32)
Other					
Drinking water source					
Improved	1071 (19.15)	1448 (57.17)	2923 (43.04)	1571 (49.38)	7013 (42.19)
Unimproved	3539 (80.85)	1178 (42.83)	3502 (56.96)	1459 (50.62)	9678 (57.82)
Number of living children					
1–3	2372 (50.73)	1201 (43.25)	3087 (47.59)	1482 (47.31)	8142 (47.22)
4–6	1691 (36.11)	1050 (40.32)	2472 (37.99)	1111 (36.6)	6324 (37.76)
>6	547 (13.16)	375 (16.43)	866 (14.42)	437 (16.09)	2225 (15.03)
Religion					
Orthodox	1856 (48.51)	912 (38.47)	1688 (34.50)	723 (28.84)	5179 (37.58)
Protestant	704 (17.96)	512 (21.67)	1422 (25.73)	584 (22.73)	3222 (22.02)
Muslim	1838 (28.77)	1112 (36.59)	3185 (37.53)	1660 (45.72)	7795 (37.15)
Other	212 (4.76)	90 (3.26)	130 (2.24)	63 (2.71)	495 (3.24)
Mother’s BMI					
Underweight	1210 (22.53)	621 (20.38)	1741 (20.23)	783 (20.51)	4355 (20.91)
Normal	3261 (75.01)	1902 (76.19)	4391 (75.85)	2058 (75.34)	11612 (75.60)
Overweight	117 (2.34)	81 (2.61)	227 (3.26)	145 (3.36)	570 (2.89)
Obese	22 (0.13)	22 (0.82)	66 (0.65)	44 (0.79)	154 (0.60)
Media Access					
Has no access/access <1 a week	4229 (92.84)	2336 (88.67)	5329 (79.66)	2635 (85.62)	14529 (86.70)
Has media access at least once a week	381 (7.16)	290 (11.33)	1096 (20.34)	395 (14.38)	2162 (13.30)
Place of residence					
Urban	518 (9.13)	250 (4.94)	670 (9.37)	253 (4.84)	1691 (7.07)
Rural	4092 (90.87)	2376 (95.06)	5755 (90.63)	2777 (95.16)	15000 (92.93)
Region					
Tigray	385 (4.93)	214 (5.07)	508 (4.59)	235 (4.55)	1342 (4.79)
Afar	317 (1.15)	192 (1.26)	794 (1.22)	376 (1.13)	1679 (1.19)
Amhara	720 (27.15)	348 (20.99)	701 (21.33)	295 (17.40)	2064 (21.72)
Oromia	923 (39.79)	555 (42.15)	1060 (44.31)	572 (49.84)	3110 (44.02)
Somali	308 (1.27)	206 (5.22)	572 (3.03)	466 (4.61)	1552 (3.53)
Benishangul-Gumuz	393 (1.10)	216 (1.08)	579 (1.19)	244 (0.95)	1432 (1.08)
SNNPR	759 (23.22)	474 (22.85)	1021 (23.09)	392 (20.64)	2646 (22.45)
Gambela	278 (0.24)	143 (0.29)	530 (0.36)	201 (0.20)	1152 (0.27)
Harari	227 (0.19)	134 (0.20)	295 (0.19)	142 (0.18)	798 (0.19)
Addis Ababa	100 (0.69)	47 (0.60)	59 (0.46)	18 (0.27)	224 (0.51)
Dire Dawa	200 (0.27)	97 (0.30)	306 (0.24)	89 (0.24)	692 (0.26)

Frequencies are unweighted, percentages are weighted.

*SNNPR* Southern Nations, Nationalities, and Peoples' Region.

### Trend analysis in ARI prevalence

#### Temporal Trend in ARI prevalence at the regional level

This section presents the trends in ARI prevalence between 2000 and 2016. Overall, Ethiopia showed a mixed pattern (fall and rise) in ARI prevalence among under-5 children over the study period, from 11.50% in 2000 to 10.40% in 2005, 11.80% in 2011, and 9.37% in 2016. Nationally, the largest reduction in ARI prevalence was seen in 2011–2016 with a 2.43% point (%p) change compared to the 1.10 and 1.40%p change in 2000–2005 and 2005–2011, respectively. Similarly, regions showed inconsistent trends in ARI prevalence during the study period. In 2000–2005, most regions, except for Amhara, Harari, SNNPR, and Benishangul-Gumuz, showed a reduction in ARI prevalence. The largest reductions in ARI prevalence during this period were observed in Afar, Somali, and Dire Dawa, at 12.98, 8.96, and 6.47%p, respectively. The Amhara region showed the largest increase in ARI prevalence during 2000–2005, registering a 6.96%p increase.

**Fig. 2:**
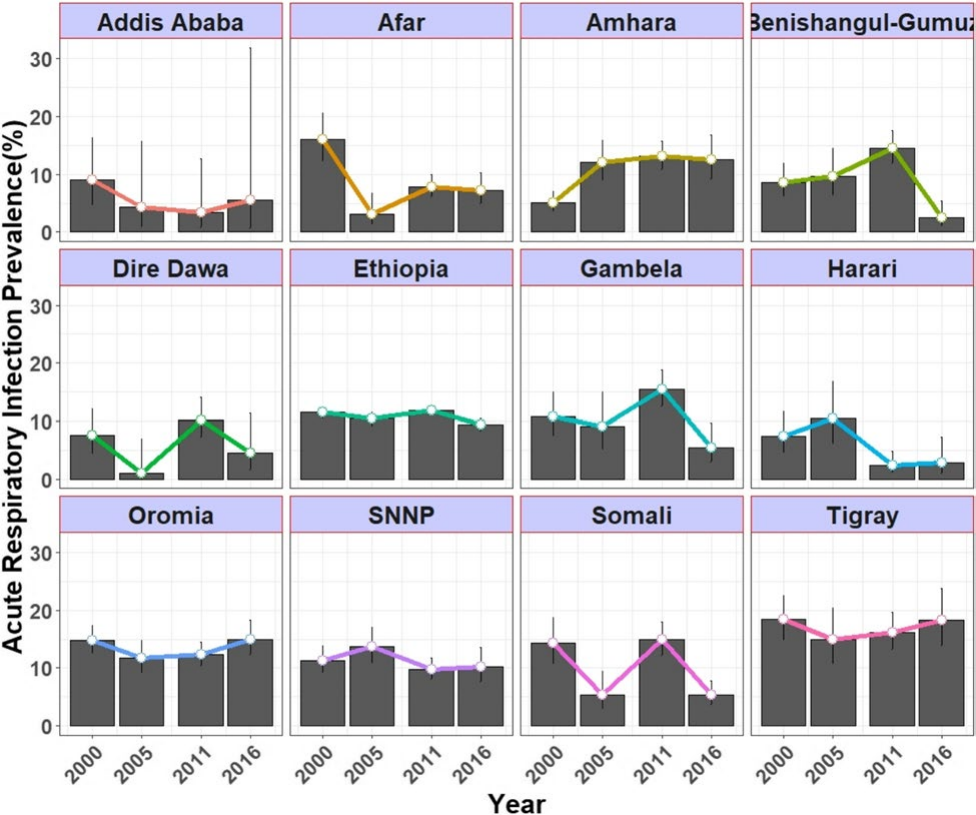
Trends in the ARI prevalence among under-5 children per region in the EDHS years 2000, 2005, 2011, and 2016

**Fig. 3:**
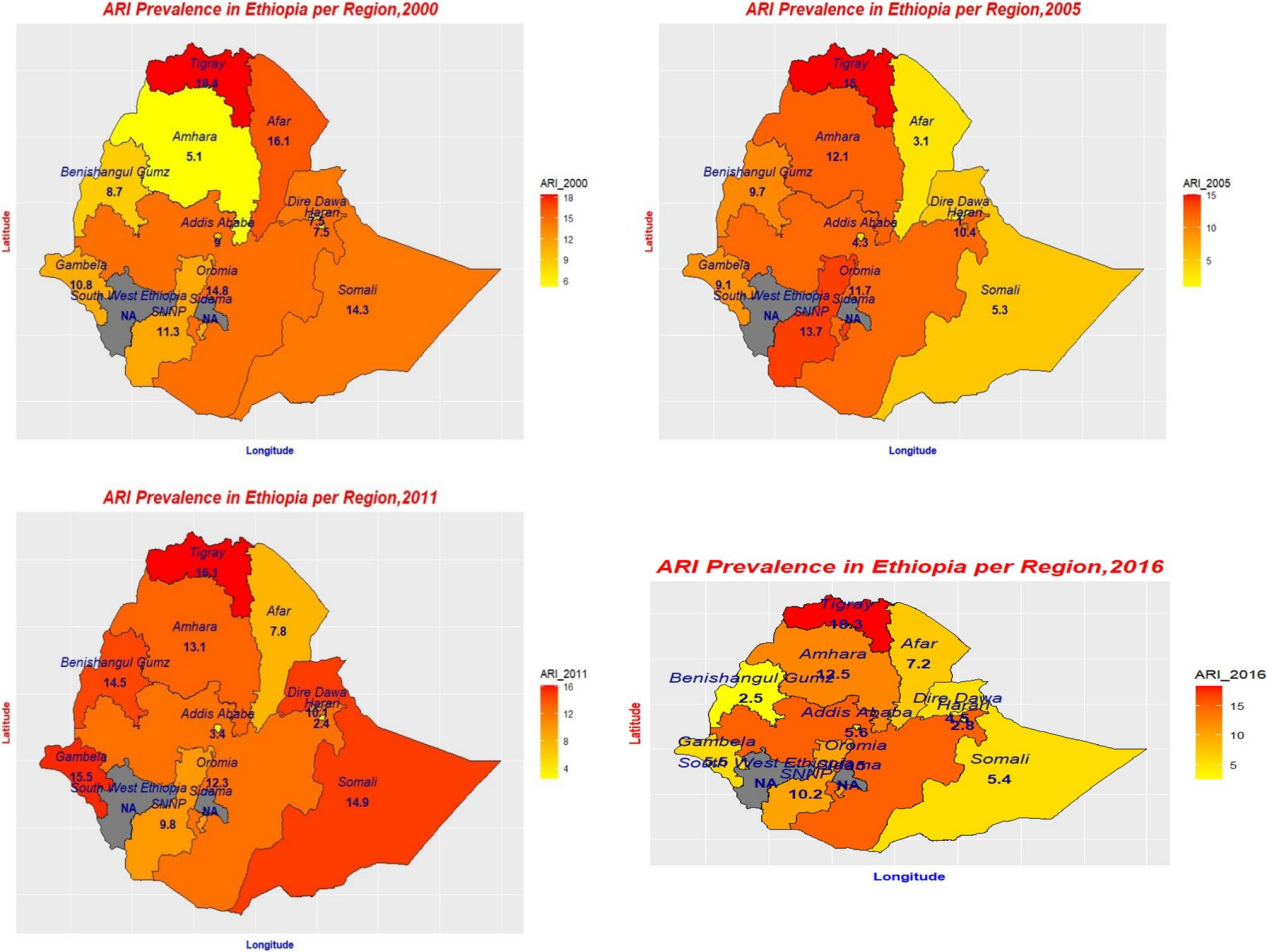
ARI prevalence by region 2000(upper left), 2005(upper right), 2011(lower left), 2016(lower right)

In 2005–2011, seven regions and one city administration showed an increase in ARI prevalence, with Somali registering the highest increase (9.56%p) among the regions. However, only three regions, Harari, SNNPR, and Addis Ababa, showed reductions in ARI prevalence of 8.03, 3.91, and 0.87%p, respectively. During 2011–2016, Benishangul-Gumuz, Gambela, Somali, Dire Dawa, Afar, and Amhara showed a reduction in ARI prevalence, whereas the rest showed an increase. Between 2011 and 2016, Benishangul-Gumuz, Gambela, and Somali ranked first to third in terms of ARI prevalence reduction, registering 12.04, 10.03, and 9.54%p, respectively. Overall, most of the regions, including Tigray, except Oromia and Amhara, showed a reduction in ARI prevalence between 2000 and 2016. Although the Tigray region showed the highest ARI prevalence in every survey year, there was a reduction in ARI prevalence over the study period. The Somlai, Afar, and Benishangul-Gumuz regions showed the highest average reduction in the study period, with 8.94%p, 8.92%p, and 6.19%p changes, respectively (Fig. 2; Fig. 3).

**Table 2:** Trends in the prevalence of ARI among under-5 children by selected characteristics, based on 2000, 2005, 2011, and 2016 EDHS

Characteristics	ARI prevalence (%) (95% CI)				ARI prevalence (%*P*) change (95% CI)			
	2000	2005	2011	2016	Phase I (2005 − 2000)	Phase II (2011 − 2005)	Phase III (2016 − 2011)	Overall (2016 − 2000)
Child age								
< 12 months	12.70(10.30,15.50)	14.10(10.70,18.50)	13.70(12.30,15.30)	9.52(7.60,11.90)	1.4(0.77,2.03)	−0.4(−1.01,0.21)	−4.18(−4.63,−3.73)	−3.18 (−3.49, −2.87)
12–23 months	15.30(13.00,17.90)	13.60(10.80,17.00)	0	10.50(8.80,12.60)	−1.7(−2.36,−1.04)	−13.6(−14.10,13.102)	10.5(10.14,10.86)	−4.80 (−5.17, −4.43)
24–35 months	12.40(10.40,14.60)	9.39(7.21,12.20)	13.40(11.70,15.30)	8.35(6.94,10.00)	−3.01(−3.49,−2.53)	4.01(3.55,4.47)	−5.05(−5.47,−4.63)	−4.05 (−4.35, −3.75)
36–47 months	10.20(8.58,12.10)	9.42(7.41,11.90)	10.50(9.10,12.10)	0	−0.78(−1.22,−0.34)	1.08(0.66,1.50)	−10.5(−10.74,−10.26)	−10.20 (−10.40, −10.00)
48–59 months	8.77(7.24,10.60)	7.99(6.14,10.30)	9.09(7.74,10.70)	0	−0.78(−1.15,−0.41)	1.1(0.75,1.45)	−9.09(−9.30,−8.88)	−8.77 (−8.94, −8.60)
Child sex								
Male	11.30(10.00,12.60)	9.89(8.40,11.60)	11.20(10.10,12.30)	9.84(8.45,11.40)	−1.41(−1.88,−0.94)	1.31(0.87,1.75)	−1.36(−1.78,−0.94)	−1.46 (−1.76, −1.16)
Female	11.80(10.50,13.20)	10.90(9.26,12.70)	12.40(11.30,13.70)	8.89(7.55,10.40)	−0.9(−1.41,−0.39)	1.5(1.01,1.99)	−3.51(−3.93,−3.09)	−2.91 (−3.20, −2.62)
Wasting								
No	11.30(10.50,16.30)	10.20(8.43,15.90)	11.20(13.90,19.70)	9.20(7.85,13.70)	−1.1(−1.58,−0.62)	1(0.55,1.45)	−2(−2.40,−1.60)	−2.10 (−2.39, −1.81)
Yes	13.10(10.40,12.30)	11.60(9.03,11.50)	16.60(10.50,12.10)	10.40(8.15,10.40)	−1.5(−2.06,−0.94)	5(4.42,5.58)	−6.2(−6.73,−5.67)	−2.70 (−3.03, −2.37)
Mothers educational level								
No education	11.70(10.80,12.80)	10.40(9.17,11.80)	11.60(10.70,12.50)	9.27(8.10,10.60)	−1.3(−1.80,−0.80)	1.2(0.73,1.67)	−2.33(−2.74,−1.92)	−2.43 (−2.73, −2.13)
Primary	11.40(8.91,14.40)	11.80(8.95,15.40)	12.80(11.20,14.60)	10.70(8.68,13.10)	0.4(−0.13,0.93)	1(0.47,1.53)	−2.1(−2.57,−1.63)	−0.70 (−1.01, −0.39)
Secondary & higher	8.18(5.21,12.60)	4.96(2.24,10.60)	9.14(5.67,14.40)	5.00(2.62,9.35)	−3.22(−3.50,−2.94)	4.18(3.91,4.45)	−4.14(−4.40,−3.88)	−3.18 (−3.36, −3.00)
Marital Status								
Never in Union	19.00(7.15,41.80)	0	4.35(0.58,26.10)	5.00(0.66,29.30)	−19(−19.53,−18.47)	4.35(4.26,4.44)	0.65(0.47,0.83)	−14.00 (−14.40, −13.60)
In a Union	11.60(10.70,12.60)	10.50(9.36,11.80)	11.70(11.00,12.60)	9.42(8.40,10.50)	−1.1(−1.60,−0.60)	1.2(0.73,1.67)	−2.28(−2.70,−1.86)	−2.18 (−2.48, −1.88)
Not in a Union	10.10(7.12,14.00)	8.67(5.09,14.40)	12.80(9.89,16.40)	9.02(5.18,15.20)	−1.43(−1.85,−1.01)	4.13(3.70,4.56)	−3.78(−4.21,−3.35)	−1.08 (−1.35, −0.81)
Type of cooking fuel								
Electricity/LPG/Natural gas/Biogas	14.3(1.66,62.10)	0	0	7.14(2.29,20.20)	−14.3(−14.70,−13.90)	0(0.00,0.002)	7.14(6.90,7.38)	−7.16 (−7.48, −6.84)
Kerosene	9.74(5.95,15.60)	3.85(0.95,14.30)	5.88(1.44,21.00)	0	−5.89(−6.18,−5.60)	2.03(1.85,2.21)	−5.88(−6.01,−5.75)	−9.74 (−9.93, −9.55)
Coal/lignite/charcoal	7.59(3.43,16.00)	4.76(1.17,17.40)	11.2(7.65,16.00)	7.3(3.96,13.10)	−2.83(−3.09,−2.57)	6.44(6.13,6.75)	−3.9(−4.26,−3.54)	−0.29 (−0.49, −0.09)
Firewood/straw	11.8(10.80,12.90)	10.7(9.50,12.00)	11.9(11.10,12.80)	9.44(8.40,10.60)	−1.1(−1.61,−0.59)	1.2(0.72,1.68)	−2.46(−2.88,−2.04)	−2.36 (−2.66, −2.06)
Dung	10.3(7.81,13.40)	9.76(5.61,16.40)	10.4(6.78,15.50)	11.6(6.51,19.80)	−0.54(−0.99,−0.09)	0.64(0.21,1.07)	1.2(0.74,1.66)	1.30 (−0.99, 1.61)
Other								
Drinking water source								
Improved	9.15(7.56,11.00)	10.80(9.34,12.60)	11.40(10.30,12.60)	9.23(7.89,10.80)	1.65(1.18,2.12)	0.6(0.12,1.08)	−2.17(−2.58,−1.76)	0.08 (−0.23, 0.39)
Unimproved	12.20(11.20,13.40)	9.76(8.19,11.60)	12.10(11.10,13.20)	9.53(8.12,11.10)	−2.44(−2.93,−1.95)	2.34(1.89,2.79)	−2.57(−3.00,−2.14)	−2.67 (−2.67, −2.67)
1-3	12.10(10.90,13.50)	11.20(9.50,13.10)	12.30(11.20,13.60)	10.10(8.68,11.80)	-0.9(-1.43,-0.37)	1.1(0.60,1.60)	-2.2(-2.65,-1.75)	-2.00 (-2.28, -1.72)
4-6	11.00(9.59,12.60)	10.00(8.32,12.00)	11.20(9.98,12.50)	8.73(7.21,10.50)	-1(-1.47,-0.53)	1.2(0.75,1.65)	-2.47(-2.86,-2.08)	-2.27 (-2.53, -2.01)
>6	10.40(8.12,13.30)	8.80(6.32,12.10)	11.50(9.58,13.90)	8.47(6.19,11.50)	-1.6(-2.03,-1.17)	2.7(2.28,3.12)	-3.03(-3.42,-2.64)	-1.93 (-1.93, -1.93)
Religion								
Orthodox	9.54(8.28,11.00)	11.20(9.29,13.40)	14.30(12.70,16.10)	12.40(10.20,15.10)	1.66(1.18,2.14)	3.1(2.57,3.63)	-1.9(-2.44,-1.36)	2.86 (-2.57, 3.15)
Protestant	11.80(9.61,14.40)	13.90(11.10,17.10)	11.20(9.64,12.90)	9.08(7.00,11.70)	2.1(1.49,2.71)	-2.7(-3.27,-2.13)	-2.12(-2.52,-1.72)	-2.72 (-3.02, -2.42)
Muslim	12.80(11.40,14.40)	8.27(6.79,10.00)	10.80(9.80,12.00)	8.19(6.97,9.61)	-4.53(-4.99,-4.07)	2.53(2.14,2.92)	-2.61(-2.98,-2.24)	-4.61 (-4.98, -4.24)
Other	16.50(12.10,22.10)	7.78(3.74,15.50)	8.46(4.74,14.70)	7.94(3.32,17.80)	-8.72(-9.26,-8.18)	0.68(0.34,1.02)	-0.52(-0.85,-0.19)	-8.56 (-8.56, -8.56)
Mothers BMI								
Underweight	12.20(10.50,14.20)	9.50(7.43,12.10)	13.20(11.60,14.80)	10.60(8.63,13.00)	-2.7(-3.18,-2.22)	3.7(3.24,4.16)	-2.6(-3.07,-2.13)	-1.60 (-1.89, -1.31)
Normal	11.40(10.30,12.50)	10.90(9.56,12.40)	11.20(10.30,12.20)	9.18(8.01,10.50)	-0.5(-1.01,0.01)	0.3(-0.18,0.78)	-2.02(-2.42,-1.62)	-2.22 (-2.46, -1.98)
Overweight	9.40(5.27,16.20)	7.41(3.35,15.60)	11.00(7.54,15.80)	7.59(4.24,13.20)	-1.99(-2.36,-1.62)	3.59(3.22,3.96)	-3.41(-3.77,-3.05)	-1.81 (-1.90, -1.72)
Obese	4.55(0.61,27.10)	0	15.20(8.31,26.00)	2.27(0.31,14.70)	-4.55(-4.67,-4.43)	15.2(14.84,15.56)	-12.93(-13.29,-12.57)	-2.28 (-2.28, -2.28)
Media Access								
Has no access/access <1 a week	11.80(10.80,12.80)	10.20(9.02,11.50)	11.90(11.00,12.80)	9.37(8.32,10.50)	-1.6(-2.09,-1.11)	1.7(1.24,2.16)	-2.53(-2.95,-2.11)	-2.43 (-2.68, -2.18)
Has media access at least once a week	8.66(6.22,11.90)	11.70(8.49,16.00)	11.40(9.65,13.40)	9.37(6.86,12.70)	3.04(2.55,3.53)	-0.3(-0.80,0.20)	-2.03(-2.44,-1.62)	0.71 (-0.71, 0.71)
Place of residence								
Urban	8.30(6.21,11.00)	3.60(1.88,6.78)	10.40(8.35,13.00)	5.93(3.60,9.61)	-4.7(-4.95,-4.45)	6.8(6.53,7.07)	-4.47(-4.78,-4.16)	-2.37 (-2.67, -2.07)
Rural	11.90(11.00,13.00)	11.10(9.87,12.40)	11.90(11.10,12.80)	9.69(8.64,10.80)	-0.8(-1.32,-0.28)	0.8(0.31,1.29)	-2.21(-2.64,-1.78)	-2.21 (-2.21, -2.21)

Note: No education: those who did not attend school; Primary: those who completed grades 1–8; and Secondary and higher: those who completed grades 9–12, and those with a college certificate, diploma, or above. Improved water: household’s drinking water source is from protected spring, protected well, or piped water, and Unimproved water source: household’s drinking water source is from unprotected spring, unprotected well, tanker truck, surface water, irrigation channel, or cart with small truck. Types of cooking fuel are based on EDHS category.

Prevalence of ARI per region for each year are illustrated in Fig. 3.

#### Trend in ARI prevalence by children characteristics

The trend in ARI prevalence in under-5 children showed fluctuation (rise and fall) according to their characteristics. Between 2000 and 2016, major reductions in the prevalence of ARI were observed in some categories (Table 2).

**Table 3:** Multilevel mixed-effect logistic regression analysis of individual-, household-, and cluster-level factors associated with ARI among under-5 children in Ethiopia, 2000–2016

Characteristics fixed effect	Null Model	Model I AOR (95% CI)		Model II AOR (95%CI)	Model III AOR (95%CI)	
Child age						
< 12 months (ref)		1			1	
12–23 months		0.88 (0.74, 1.04)			0.89 (0.75, 1.06)	
24–35 months		0.68 (0.59, 0.79)	***		0.68 (0.59, 0.79)	***
36–47 months		0.66 (0.56, 0.77)	***		0.65 (0.55, 0.76)	***
48–59 months		0.52 (0.44, 0.61)	***		0.51 (0.44, 0.61)	***
Child vaccination						
No (ref)		1			1	
Yes		1.06 (0.94, 1.20)			1.07 (0.94, 1.21)	
Mothers educational level						
No education (ref)		1			1	
Primary		1.07 (0.94, 1.22)			1.08 (0.95, 1.23)	
Secondary & higher		0.70 (0.49, 1.00)	*		0.79 (0.55, 1.13)	
Marital Status						
Never in a union (ref)		1			1	
In a Union		1.16 (0.53, 2.57)			1.04 (0.48, 2.27)	
Not in a Union		1.20 (0.53, 2.70)			1.08 (0.48, 2.42)	
Mother’s BMI						
Underweight (ref)		1			1	
Normal		0.93 (0.82, 1.05)			0.93 (0.83, 1.06)	
Overweight		1.08 (0.78, 1.48)			1.11 (0.81, 1.53)	
Obese		1.49 (0.76, 2.91)			1.54 (0.78, 3.01)	
Wasting						
No (ref)		1			1	
Yes		1.20 (1.02, 1.41)^*^			1.18 (1.00, 1.39)^*^	
Type of cooking fuel						
Electricity/LPG/Natural gas/Biogas (ref)		1			1	
Kerosene		0.20 (0.02, 1.69)			0.22 (0.02, 1.91)	
Coal/lignite/charcoal		0.60 (0.20, 1.82)			0.57 (0.19, 1.74)	
Firewood/straw		0.84 (0.30, 2.31)			0.64 (0.23, 1.79)	
Dung		0.68 (0.24, 1.92)			0.51 (0.18, 1.47)	
Number of living children						
1-3 (ref)		1			1	
4-6		0.84 (0.75, 0.94)	**		0.84 (0.75, 0.94)	**
>6		0.69 (0.59, 0.81)	***		0.69 (0.59, 0.81)	***
Religion						
Orthodox (ref)		1			1	
Protestant		1.07 (0.90, 1.28)			1.06 (0.89, 1.27)	
Muslim		1.05 (0.89, 1.24)			1.04 (0.88, 1.24)	
Other		1.21 (0.89, 1.64)			1.19 (0.88, 1.62)	
Place of residence						
Urban (ref)				1	1	
Rural				1.57 (1.00, 2.45)	1.54 (0.97, 2.45)	
Media Access						
Has no access/access <1 a week (ref)				1	1	
Has media access at least once a week				1.07 (0.88, 1.29)	1.07 (0.88, 1.31)	
Access to electricity						
No				1	1	
Yes				0.79 (0.54, 1.15)	0.81 (0.55, 1.19)	

### Individual- and cluster-level factors influencing ARI prevalence among under-5 children

#### Factors influencing ARI prevalence among under-5 children

Table 3 presents the multilevel mixed-effects logistic regression models used to investigate the relationships between individual-, household- and cluster-level factors and ARI symptoms. Model I showed that the individual- and household-level variables including child’s age, mother’s education level, wasting, and number of living children were significantly associated with ARI symptoms. Model II showed that place of residence, a cluster-level variable, was significantly associated with ARI symptoms. Model III, with individual-, household- and cluster-level variables, indicated that child’s age, wasting, number of living children, and place of residence had statistically significant effects on ARI symptoms. Using AIC as a means to determine model fit, the last model (Model III) was found to be the best-fit model (AIC = 12,335.65).

**Table 4.** Multilevel mixed-effect logistic regression analysis of individual-, household-, and cluster-level factors associated with ARI among under-5 children in Ethiopia, 2000–2016 (continued).

Characteristics fixed effect	Null Model	Model I AOR (95% CI)		Model II AOR (95%CI)		Model III AOR (95%CI)	
Survey Year							
2000				1		1	
2005				1.00 (0.79, 1.27)		1.00 (0.78, 1.27)	
2011				1.12 (0.90, 1.40)		1.05 (0.84, 1.32)	
2016				1.11 (0.87, 1.41)		0.89 (0.69, 1.14)	
AIC	12434.25	12336.36		12428.36		12335.65	
BIC	12457.42	12529.43		12497.87		12575.05	
Log Likelihood	−6214.13	−6143.18		−6205.18		−6136.82	
Num. obs.	16,691	16,691		16,691		16,691	
Num. groups: V021:Survey_Year	2059	2059		2059		2059	
Num. groups: Survey_Year	4	4		4		4	
Var: V021:Survey_Year (Intercept)	0.86	0.87		0.86		0.88	
Var: Survey_Year (Intercept)	0.00	0.00		0.00		0.00	

The Null Model includes no explanatory variables, Model I includes the individual- and household-level variables, Model II includes the cluster-level variables, Model III includes the individual-, household- and cluster-level variables

*AOR* Adjusted odds ratio, *CI* confidence interval, *SNNPR* Southern Nations, Nationalities, and Peoples’ Region

Significance codes: 0 ‘***’ 0.001 ‘**’ 0.01 ‘*’ 0.05 ‘.’ 0.1 ‘ ’ 1, ref = reference category

As a result, in comparison to children aged below12 months, children who were 24–35, 36–47, and 48–59 months old had lesser odds of developing ARI with an adjusted odds ratio [AOR] = 0.68 (95% confidence interval [CI]: 0.59–0.79, *p* < 0.001), 0.65 (0.55–0.76, *p* < 0.001), and 0.51 (0.44–0.61, *p* < 0.001), respectively. Regarding the nutritional status of children, those who were wasted were more likely to develop ARI than those who were not wasted with an AOR of 1.18 (95% CI: 1.00–1.39, *p* = 0.04). The number of living children in the family is the other factor found to be determinant; children who had 4–6 siblings had an AOR of 0.84 (95% CI: 0.75–0.94, *p* = 0.002) to develop ARI than those who had 1–2 siblings. While those who had more than six siblings had AOR of 0.69 (95%CI: 0.59–0.81, *p* < 0.001) to develop ARI than those who had 1–2 siblings. Among the cluster-level factors, only place of residence was found to be significant.

## Discussion

Our findings revealed an inconsistent trend in ARI prevalence across regions in Ethiopia between 2000 and 2016. During 2000–2005, the majority of regions showed a reduction in ARI prevalence. The three largest reductions in ARI prevalence were observed in the Afar, Somali, and Dire Dawa populations. During 2005–2011, except for Harari, SNNPR, and Addis Ababa, all regions and the Dire Dawa city administration showed an increase in ARI prevalence. From 2011 to 2016, the Benishangul-Gumuz, Gambela, Somali, Afar, and Amhara regions and Dire Dawa city administration showed a reduction in ARI prevalence, whereas the remaining regions and Addis Ababa city administration showed an increase in ARI prevalence. A possible reason for this unequal risk of exposure to ARI could be socioeconomic inequality across communities and regions [26, 27]. This could also be due to the geographical location of high-risk areas of the country being located in highland areas or because most households in these areas relied on cow dung or charcoal for heat, and many children were under one year of age [28]. It may also be related to geographical differences in the availability of health services, lack of a sufficient and safe supply of drinking water, variations in food intake, substandard household characteristics, and inadequate latrine facilities, all of which could promote the spread of infections [29–32].

In addition, the inconsistent temporal trend in ARI prevalence per region could be caused by environmental, climatic, infrastructural, and altitudinal differences across regions [33]. It could also be due to disparities in the density of health workers and provision of services [34]. The changes in temporal trend of the prevalence of ARI could be influenced by seasonal variations. The incidence of ARI symptoms is reportedly higher in rural than in urban areas during the dry season [35, 36]. Furthermore, environmental quality, including air pollution, and population density could impact the regional difference in ARI prevalence in under-5 children [37]. We also found regional variation in ARI prevalence among under-5 children in different survey years. We found that ARI prevalence was the highest in Tigray during the four consecutive survey years. Oromia, Amhara, SNNPR, Afar, Gambela, and Somali also showed a relatively higher ARI prevalence than the other regions, whereas a lower ARI prevalence was recorded in Addis Ababa, Harari, Dire Dawa, and Benishangul-Gumuz. The Afar and Amhara regions showed the lowest and largest ARI prevalence, respectively, during this period.

Moreover, our study found that older children had lower odds of acquiring ARI than children aged < 12 months. Similar results have been reported in studies conducted in Ethiopia [38, 39]. A possible reason could be that the older the age is of these children, the more equipped the immune system is to fight infections, including ARI. However, a study conducted in SSA countries found the opposite result [55]. It stated that children aged 48–59 months had 1.19 times higher odds of experiencing ARIs than those aged below 12 months. Additionally, a study in Malawi found no significant association between child age and ARI [56].

Our study did not find any significant association between the prevalence of ARI among under 5 children and vaccination. A similar result was found in a study conducted in Ethiopia [6], while contrasting result was found in a study conducted in Bangladesh [42]. Another study in Ethiopia found a statistically non-significant negative association between vaccines and the risk of ARI [35]. The possible reason for the negative association between ARI and vaccination could be that atypical bacteria are common causes of ARI [43]. A study in Morocco showed that viruses are the most common cause of ARIs in preschool children [44]. Despite the fact that vaccines have played an essential role in preventing bacterial respiratory infections, there are non-vaccine pneumococcal serotypes that cause ARIs [45]. Common childhood vaccines like PCV are effective in preventing respiratory infections caused by Streptococcus pneumonia, H-influenza, and measles [46]. In 2020 it was estimated that PCV 10/13 prevents 23.8 million episodes of antibiotic-treated ARIs in children aged 24 to 59 months in LMICs [47]. In Ethiopia the PCV-10 vaccine was introduced in late 2011 to prevent severe forms of pneumococcal disease such as pneumonia and meningitis which were previously estimated to account for up to 28% of all deaths among children under 5 [48]. Therefore, the significant reduction in ARI might be at least partially attributable to the PCV-10 vaccine. Our study did not find out any significant association between the prevalence of ARI and maternal education. Similar results were reported in previous studies [49–52]. The absence of a correlation between maternal education and ARI could be explained by the fact that many risk factors are required for ARI development. Consequently, some of these risk factors (possible confounders) were not controlled in our study. With this regard, further epidemiological studies must be conducted. In addition, our study found no significant associations between ARI and sex of the children. Similar results have been obtained in other studies [53–55]. It remains unclear how sex affects the incidence of ARI (community-acquired pneumonia) in children [56, 57], and further studies are required to fully understand the sexual differences in the risk of childhood ARI. Regarding the nutritional status of children, children who were wasted were more likely to develop ARI than those who were not wasted. Similar results were reported in other studies [39, 40, 42]. Moreover, the cluster-level variable “place of residence” living in urban versus rural was found to be the other determining factor of ARI prevalence in line with other studies [21, 58]. Children living in rural areas were more likely to be exposed to ARI than those living in urban areas. Overall, no consistent pattern among the regions was observed in the temporal trend of ARI prevalence in under-5 children. During the periods of 2000–2005 and 2011–2016, five regions and the two city administrations showed a reduction in ARI prevalence. However, from 2005 to 2011, only two regions and Addis Ababa city administration showed a reduction, while the rest showed an increase in ARI prevalence.

The findings of this study should help take appropriate policy directions and interventions to prevent ARI. Enhancing socioeconomic conditions, infrastructure, nutrition, and healthcare services, especially in underprivileged areas, is important for the early detection and management of respiratory infections. In addition, it is essential to consider regional variations that encourage the development and implementation of integrated healthcare policies that address both the preventive and curative aspects of ARI. Our study had some limitations. First, because the symptoms of ARI were self-reported without being measured clinically, the variables were subject to reporting bias. Secondly, household hygiene practices, which are significant predictors of infectious diseases in people of all ages, were not included in the data.

## Conclusion

This study indicates that there is an inconsistent trend in ARI prevalence at both the regional and national levels in Ethiopia. The temporal trend displayed a mixed pattern (decreasing and increasing). Between 2000 and 2005 and 2011–2016, most regions experienced a reduction in ARI prevalence, whereas an increase was observed during 2005–2011. The study revealed regional variations in the temporal trend of ARI prevalence among under-5 children across Ethiopia. In addition, the results showed that children aged > 12 months were less likely to develop ARI than those aged < 12 months. Children who were wasted were more likely to develop ARI than those who were not wasted. Children living in urban areas were less likely to develop ARI than those in the reference group. Moreover, between 2000 and 2016, the trend analysis showed that, the risk of developing acute respiratory infection among children aged under 5 years reduced in Ethiopia. The prevalence of ARI could be reduced through the implementation of various interventions aimed at improving the nutritional status of children and the socioeconomic status and infrastructure of the less developed regions of the country. As we observed that mother’s education, and child’s sex were not associated with ARI, there is a need to gain a more detailed understanding of these potential risk factors for ARI.

## Supplementary Information

Supplementary Material 1.

## Data Availability

N/A (We used publicly available data.)
