# Supplementary material for: Trends in acute respiratory infection and associated factors among under-5 children in Ethiopia
Source: BMC Pediatr. 2026 Jan 19;26:107. doi: 10.1186/s12887-025-06100-x (PMC12895734; doi:10.1186/s12887-025-06100-x)
Supplement: Supplementary file 1 — Supplementary Material 1. [file 12887_2025_6100_MOESM1_ESM.docx]

**Text S1. Trend in ARI prevalence by background characteristics of children**

The trend in ARI prevalence among under-5 children showed fluctuation (rise and fall) according to their characteristics. With respect to child age, the greatest decrease in ARI prevalence is observed in children aged 36-47 months, followed by those who are in the age group 48-59 months. Concerning the type of the cooking fuel used by children household, children whose household used kerosene showed the largest reduction (3.25%p) in ARI prevalence followed by those who belonged to a household that use Electricity/LPG/Natural gas/Biogas as source of cooking fuel with a 2.39%p.

Moreover, regarding drinking water source children who have access to unimproved water source showed the greater reduction with a 0.89%p while their counter parts showed an average rise (0.03%p) to ARI prevalence in the study period. Between the year 2000-2016 children who lived in urban areas showed a larger reduction in ARI prevalence with an average 0.74%p while those who lived in rural areas showed a 0.74%p change (Table S1).

**Table S1. Trends in the prevalence of ARIs among under-5 children by selected characteristics 2000,2005,2011, and 2016 Ethiopian Demographic Health Survey.**

| **Characteristics** | **Percentage** | | | | **Percentage point change in ARI prevalence** | | | |
| --- | --- | --- | --- | --- | --- | --- | --- | --- |
|  | **2000** | **2005** | **2011** | **2016** | **2005 - 2000** | **2011 - 2005** | **2016 - 2011** | **Overall (Average)** |
| **Child age** |  |  |  |  |  |  |  |  |
| < 12 months | 12.70 | 14.10 | 13.70 | 9.52 | 1.40 | -0.40 | -4.18 | -1.06 |
| 12 - 23 months | 15.30 | 13.60 |  | 10.50 | -1.70 | -13.60 | 10.50 | -1.60 |
| 24 - 35 months | 12.40 | 9.39 | 13.40 | 8.35 | -3.01 | 4.01 | -5.05 | -1.35 |
| 36 - 47 months | 10.20 | 9.42 | 10.50 |  | -0.78 | 1.08 | -10.50 | -3.40 |
| 48 - 59 months | 8.77 | 7.99 | 9.09 |  | -0.78 | 1.10 | -9.09 | -2.92 |
| **Child sex** |  |  |  |  |  |  |  |  |
| Male | 11.30 | 9.89 | 11.20 | 9.84 | -1.41 | 1.31 | -1.36 | -0.49 |
| Female | 11.80 | 10.90 | 12.40 | 8.89 | -0.90 | 1.50 | -3.51 | -0.97 |
| **Wasting** |  |  |  |  |  |  |  |  |
| Yes | 13.10 | 11.60 | 16.60 | 10.40 | -1.50 | 5.00 | -6.20 | -0.90 |
| No | 11.30 | 10.20 | 11.20 | 9.20 | -1.10 | 1.00 | -2.00 | -0.70 |
| **Mothers educational level** |  |  |  |  |  |  |  |  |
| No education | 11.70 | 10.40 | 11.60 | 9.27 | -1.30 | 1.20 | -2.33 | -0.81 |
| Primary | 11.40 | 11.80 | 12.80 | 10.70 | 0.40 | 1.00 | -2.10 | -0.23 |
| Secondary & higher | 8.18 | 4.96 | 9.14 | 5.00 | -3.22 | 4.18 | -4.14 | -1.06 |
| **Marital Status** |  |  |  |  |  |  |  |  |
| Never in Union | 19.00 |  | 4.35 | 5.00 | -19.00 | 4.35 | 0.65 | -4.67 |
| In a Union | 11.60 | 10.50 | 11.70 | 9.42 | -1.10 | 1.20 | -2.28 | -0.73 |
| Not in a Union | 10.10 | 8.67 | 12.80 | 9.02 | -1.43 | 4.13 | -3.78 | -0.36 |
| **Type of cooking fuel** |  |  |  |  |  |  |  |  |
| Electricity/LPG/Natural gas/Biogas | 14.3 |  |  | 7.14 | -14.3 | 0.00 | 7.14 | -2.39 |
| Kerosene | 9.74 | 3.85 | 5.88 |  | -5.89 | 2.03 | -5.88 | -3.25 |
| Coal/lignite/charcoal | 7.59 | 4.76 | 11.2 | 7.3 | -2.83 | 6.44 | -3.9 | -0.10 |
| Firewood/straw | 11.8 | 10.7 | 11.9 | 9.44 | -1.10 | 1.20 | -2.46 | -0.79 |
| Dung | 10.3 | 9.76 | 10.4 | 11.6 | -0.54 | 0.64 | 1.2 | 0.43 |
| Other |  |  |  |  |  |  |  |  |
| **Drinking water source** |  |  |  |  |  |  |  |  |
| Improved | 9.15 | 10.80 | 11.40 | 9.23 | 1.65 | 0.60 | -2.17 | 0.03 |
| Unimproved | 12.20 | 9.76 | 12.10 | 9.53 | -2.44 | 2.34 | -2.57 | -0.89 |

**Table S1. Continued**

| **Characteristics** | **Percentage** | | | | **Percentage point change in ARI prevalence** | | | |
| --- | --- | --- | --- | --- | --- | --- | --- | --- |
|  | **2000** | **2005** | **2011** | **2016** | **2005 - 2000** | **2011 - 2005** | **2016 - 2011** | **Overall (Average)** |
| **Number of living children** |  |  |  |  |  |  |  |  |
| 1-3 | 12.10 | 11.20 | 12.30 | 10.10 | -0.90 | 1.10 | -2.20 | -0.67 |
| 4-6 | 11.00 | 10.00 | 11.20 | 8.73 | -1.00 | 1.20 | -2.47 | -0.76 |
| >6 | 10.40 | 8.80 | 11.50 | 8.47 | -1.60 | 2.70 | -3.03 | -0.64 |
| **Religion** |  |  |  |  |  |  |  |  |
| Orthodox | 9.54 | 11.20 | 14.30 | 12.40 | 1.66 | 3.10 | -1.90 | 0.95 |
| Protestant | 11.80 | 13.90 | 11.20 | 9.08 | 2.10 | -2.70 | -2.12 | -0.91 |
| Muslim | 12.80 | 8.27 | 10.80 | 8.19 | -4.53 | 2.53 | -2.61 | -1.54 |
| Other | 16.50 | 7.78 | 8.46 | 7.94 | -8.72 | 0.68 | -0.52 | -2.85 |
| **Mothers BMI** |  |  |  |  |  |  |  |  |
| Underweight | 12.20 | 9.50 | 13.20 | 10.60 | -2.70 | 3.70 | -2.60 | -0.53 |
| Normal | 11.40 | 10.90 | 11.20 | 9.18 | -0.50 | 0.30 | -2.02 | -0.74 |
| Overweight | 9.40 | 7.41 | 11.00 | 7.59 | -1.99 | 3.59 | -3.41 | -0.60 |
| Obese | 4.55 |  | 15.20 | 2.27 | -4.55 | 15.20 | -12.93 | -0.76 |
| **Media Access** |  |  |  |  |  |  |  |  |
| Has no access/access <1 a week | 11.80 | 10.20 | 11.90 | 9.37 | -1.60 | 1.70 | -2.53 | -0.81 |
| Has media access at least once a week | 8.66 | 11.70 | 11.40 | 9.37 | 3.04 | -0.30 | -2.03 | 0.24 |
| **Place of residence** |  |  |  |  |  |  |  |  |
| Urban | 8.30 | 3.60 | 10.40 | 5.93 | -4.70 | 6.80 | -4.47 | -0.79 |
| Rural | 11.90 | 11.10 | 11.90 | 9.69 | -0.80 | 0.80 | -2.21 | -0.74 |

**Note: No education: those who did not attend school, Primary: those who completed grades 1–8, Secondary and higher: those who completed grades 9–12, and those with a college certificate, diploma, or above; Improved water: household’s drinking water source is from protected spring, protected well, and piped water, Unimproved water source: household’s drinking water source is from unprotected spring, unprotected well, tanker truck, surface water, irrigation channel, and cart with small truck; type of cooking fuel is based on EDHS category.**
